# Pneumonia

**Published:** 1853-04

**Authors:** 


### ART. II.—*Pneumonia.* By SMELFUNGUS.

"During the angry controversy between the Nominalists and the Realists, certain offensive books were ordered to be chained in the libraries. It were well if, by a similar decree, nineteen-twentieths of the materia medica were locked up in the cabinets of the curious."—PROF. ABNER H. BROWN.

Smelfungus, standing a few days since on the icy bank by the little watch-house beside the railroad bridge at Portageville, upraised his hat in reverence to the mind that planned it. There stood the bridge, a structure, firm, sure, and steadfast, yet light, graceful, and symmetrical. Down hundreds of feet in the abysm flowed the dark river, fretting about the solid piers, where,

"Far, fa\i beneath the vast incumbent pile
Slept the broad rock!"

"Here," he exclaimed, "here at last is the true emblem of medical science. Every stick in that vast congeries of trestle-work is isolated, and capable, in event of decay, of removal and replacement by another. On no one timber rests any special importance. Any worthless piece may be cast aside without detriment to the unity of the whole. And so in medicine. Our temple is not built of impoverishable materials, neither does the safety of our art rest upon any one fact, medicine, or theory. In the progress of discovery our facts may become worthless, our medicines inert or hurtful, our theories 'the baseless fabric of a vision,' and still the goodly structure stands; for a new and truer fact is substituted; by a better interpretation of nature we have some better treatment of disease, and for our withered theories we gain some surer basis. No mortise or tenon is in the edifice, and the casting out of an erroneous idea does not involve the destruction of its neighbor, or endanger the stability of the whole.

"Therefore, oh watchman! scan closely all the parts — reject fearlessly all decayed and broken elements. Go on, ye builders, taking from and adding to, until the temple of Esculapius stands a perfect whole of solid and enduring material!"

Having had an easy delivery of these pregnant remarks, Smelfungus retired to the Lauman House to warm his feet and get a cigar. Once thoroughly warmed and under the influence of a specially good cigar, he went into a discussion of medical matters — things new and old — with all that homely enthusiasm for the good, and peppery indignation for the bad that characterize him.

"*In medio tutissimus ibis,* said the olden poet, and ever since, like wine long kept, the pithy adage has grown richer and more truthful. In all the little eddies and whirlpools of medical inquiry, (with little great men floating like chips upon the seething waters) we recognize two mighty currents, deep flowing and rapid in their diverse ways, uplifting huge contentious waves of difference. The one of these sweeps on with gathering force against old theories, and already it threatens the gorgeous though composite pile of our old materia medica with destruction. And this is the tide, or the gulf-stream, or if you please the all-enveloping maelstrom of natural medicine."

Here Smelfungus sticks in his allegory, and like Mr. Micawber, descending abruptly from his lofty periods, he "docks the tail of sentiment."

"In short," (growing red in the face,) "here are two separate packs of fools annoying sober men with their nonsense. Here is one class, who te l us that medicine is an invention of the enemy — who call aloud for another Hercules to turn another Alpheus through those Augean stables the apothecary shops, and sweep from the learned shelves the latin-labeled drugs as things of no account or value. Oh ye cold blooded animals who look upon a patient as an interesting specimen of natural history, and turn a deaf ear to his earnest cry for pills and potions — *the world loves medicine!* Man, quoth Cuvier, (?) is a pill-taking animal, and you who deny this first want of his nature he will not call upon.

"And yet woe to the poor patient, if in shunning the natural historian he falls upon the other extreme of unlimited control over diseased action by medicines! He will surely find himself *in extremis!* These men of blood and guts, who remove a pint of disease from a hole in the brachial vein, and a half a gallon more *per anum*, are worse than their do-nothing antagonists. They are no rare birds either. I have a dozen in my circle of acquaintance whose latest author is good old Dr. Thomas. Why sir! when my poor friend Dr. T. was coming down with consumption, we met in consultation, a half a dozen brother chips, when, as the youngest man, I was first called on for an opinion, I proposed such a mild febrifuge, anodyne, and counter-irritant course as should allay the pressing inflammatory symptoms then present, and restore the tone of the stomach, old Dr. W.'s gray hairs stood up in mingled wonder and disdain. 'Give him an emetic!' thundered the doughty old Hunker. 'He 's got the consumption, *that 's* the treatment for consumption, and *that* will do him good!' The good old gentleman evidently fancied that poor T. was going to puke up a phthisis pulmonalis! And every day, with younger men, we see this same insane notion of a routine of pills, powders, and emetics, for a disease of assimilation.

"I would to God," (and Smelfungus crushes his cigar in his righteous indignation,) "that I had the power to make these men read, learn, and inwardly digest the whole of Martyn Paine's abstrusely learned volumes!* After such a course as that I fancy they would turn to our lighter periodical literature with a relish which they do not manifest at present.

"*Quò me vehis?*

'Prone to wander, Lord I feel it!' "

sings Smelfungus in answer to a gentle hint as to the subject matter of con-

---

*"Man's inhumanity to man
Makes countless thousands mourn."

versation, "and if my text be true I have been wandering in dangerous paths. Now let us get back to this middle ground if we can find it.

"The revelations constantly making in the natural history of disease have taught all seekers after truth, that medicines are not the actual necessities we have deemed them. Yet it does not hence follow that they are *per se*, useless or injurious. One great general principle may be laid down, viz., that when we can with safety omit a medicine, it is our duty so to do. But this word safety should imply not only immunity from death, but from unnecessary suffering. I like much, (always excepting a certain timidity in its tone,) the article on pneumonia, in the last 'Braithwaite,' by Dr. R. B. Todd. Dr. Todd proposes to strike out from the list of medicines in this disease, all the weighty items, such as tartar emetic, calomel, bloodletting, etc., and to substitute for them in increased doses a medicine long used in pneumonia, but considered merely as an adjunct, viz., the acetate of ammonia given in six dram doses. Externally he makes frequent use of the turpentine stupe. Thus Dr. Todd relieves us from the use of drugs frequently hurtful and unmanageable. By some process of reasoning not very lucid, he connects this change of treatment with the idea of blood poison.

"Smelfungus will help Dr. Todd through in this matter. Perhaps this blood poison may be an excess of albumen in the fluids. We have been in the habit of calling the albuminous sputa of pneumonia the result of inflammation, but the occurrence of critical albuminuria in this disease would seem to indicate that albumen was in excess throughout the system, independent of phlogistic action. Dr. Smelfungus stands ready to receive the thanks of Dr. Todd for this explanation. But, seriously, the presence of a poison in the blood has something to do with pneumonia typhoides, and who can tell us the pathological differences between that and the acute form.

"The self-limitation of pneumonia is another idea advanced by Dr. Todd. Now I have had the good fortune to see several cases of this disease, which, from the stupidity of the friends, had no medical treatment until the occurrence of bloody expectoration in the second stage alarmed them. In all these cases, the disease had reached its acme, and was on the decline, having involved only a limited portion of the lower lobe. Now, my good Sir Hunker! these cases (three or four in number) are, so far as they go, positive contradictions to your pet notion, that an inflammation once lighted in the lung, will spread like wildfire through its whole parenchyma. It may do so, mind you, but you may safely draw the conclusion that the almost uniform departure of the inflammation at a certain point in the lung, is not *all* owing to your own skill. For if you have such unlimited control over inflammation, why can you not bring it to bear on an erysipelas, or a synovitis? As Allapod says, 'hence we view,' that you do not in every case cut short the disease with your routine of bloodletting, calomel, antimony, and blistering. *It cuts itself short*, and therein only manifests its natural tendency.

"Shall we, then, abandon bloodletting in pneumonia? Even so; for it is generally unnecessary. Not so; for cases there be when the delirious mind grows rational, the swollen countenance natural, and the choked and labored pulse grows soft and easy, from the lancet.

"Shall we abandon calomel and antimony? Again yes; and again no; for cases will occur, when every means that science can prompt or art direct, are necessary to guide and govern the lava tide of inflammation, to prevent effusion and abscess, and the whole dark array of *sequelæ*.

"And blistering;" and Smelfungus speaks tenderly as a lover of his mistress, "blisters are always good, and never disappoint us." If in all the nauseous scented armamentaria of therapeutics there is one thing that Smelfungus is willing (metaphorically speaking) to take to his heart, it is Emplastrum Catharidis! He loves his blisters as fervently as old Dr. Clysterpipe his syringes, for the old man based his claim to Christian character *on the love he bore his enemies.* Pardon the pun!

"Sir Hunker! from premises like these we predict the dawning of a milder day. Pneumonia is still, and ever will be, a disease eminently requiring the guiding hand of the physician. The old (and we may still call it the usual and authorized) treatment of pneumonia, is a club in the hands of Hercules wherewith we may deal mighty blows. But a pounding less severe will answer in a majority of cases, and we shall yet learn that a rat-trap is no better than a smaller tool for catching the 'small deer' that infest our crania.

"Listen, then, to the truthful lesson! Pneumonia is, in a majority of cases, a self-limited and little dangerous disease, but it should be closely watched, lest, as sometimes happens, the diseased action may not stop at the usual point in the lower lobe, but rage on unchecked throughout its utmost borders. And mark you, man of the lancet! He who cures a pneumonia predestined to occupy a whole lung, does a goodly thing and may congratulate himself. Here come in your whole catalogue of remedies. The God Antiphlogos alone is mighty to save!"

